# Electrospun Biomaterials from Chitosan Blends Applied as Scaffold for Tissue Regeneration

**DOI:** 10.3390/polym13071037

**Published:** 2021-03-26

**Authors:** Christian Enrique Garcia Garcia, Frédéric Bossard, Marguerite Rinaudo

**Affiliations:** 1 Departamento de Ingeniería Química, Universidad de Guadalajara, Blvd. M. García Barragán #1451, Guadalajara C.P. 44430, Jalisco, Mexico; 2 Institute of Engineering Universite, Universite Grenoble Alpes, CNRS, LRP 38000 Grenoble, France; frederic.bossard@univ-grenoble-alpes.fr; 3 Biomaterials Applications, 6 Rue Lesdiguières, 38000 Grenoble, France

**Keywords:** electrospinning, chondrocyte development, AFM, chitosan, hyaluronan, PEC, nanofiber

## Abstract

Our objective in this work was to summarize the main results obtained in processing pure chitosan and chitosan/hyaluronan complex in view of biomedical applications, taking advantage of their original properties. In addition, an electrospinning technique was selected to prepare nanofiber mats well adapted for tissue engineering in relation to the large porosity of the materials, allowing an exchange with the environment. The optimum conditions for preparation of purified and stable nanofibers in aqueous solution and phosphate buffer pH = 7.4 are described. Their mechanical properties and degree of swelling are given. Then, the prepared biomaterials are investigated to test their advantage for chondrocyte development after comparison of nanofiber mats and uniform films. For that purpose, the adhesion of cells is studied by atomic force microscopy (AFM) using single-cell force spectroscopy, showing the good adhesion of chondrocytes on chitosan. At the end, adhesion and proliferation of chondrocytes in vitro are examined and clearly show the interest of chitosan nanofiber mats compared to chitosan film for potential application in tissue engineering.

## 1. Introduction

Researchers have been working for a long time on the processing of chitosan to generate new biomaterials, especially developed for biomedical applications [1,2,3,4,5,6,7,8,9,10]. A wide range of methods has been used to produce chitosan-based materials, but, in terms of practicality and efficiency, nanofibers are frequently obtained by electrospinning [1,11,12,13]. Non-woven membranes of nanofibers are well known for their porous structures and relatively large surface area, which provide ideal materials to mimic the natural extracellular matrix (ECM) required for tissue engineering [14,15]. Moreover, they allow sustained delivery of therapeutic molecules [16]. Interfiber pores promote permeability of the fiber dressings, which further enhances the exchange of oxygen and nutrients with the outside environment and are well adapted for cell culture [5,17]. As shown before, the nanofibrous structure enhances the attachment of human osteoblasts and chondrocytes and maintains characteristic cell morphology and viability when dense film is compared to nanofibers. Then, this matrix with low fiber diameters (around 200 nm) and large porosity (density around 0.04 g/cm^3^) is of particular interest in tissue engineering for controlled drug release and tissue remodeling [18].

One advantage of chitosan, a pseudo-natural polymer, is that it becomes water soluble in acidic conditions due to –NH_2_ protonation as soon as its degree of acetylation is lower than 0.5. Then, chitosan solution processing is relatively easy in order to obtain fibers, nanofibers, films, capsules, beads, sponges, gels, powder, or tablets, as chitosan turns insoluble in neutral medium (i.e., when chitosan is in the –NH_2_ form). This polymer is often difficult to characterize but its main properties are the weight-average molecular weight (MW), the average degree of acetylation (DA), and the acetyl distribution along the chains as discussed previously [19,20]. Chitosan is an interesting biodegradable and biocompatible polymer with hemostatic properties, anti-inflammatory response, antibacterial and antifungal properties often described in the literature, and is well adapted for biological applications [11,21]. In addition, chitosan is stabilized by the H-bond network in the solid state, providing good mechanical properties under film or fibrous materials.

Chitosan has already been electrospun using different acid-based solvents [22,23]. For instance, acetic acid used at concentration from 90% down to 1% (or 0.17 M) gives good fibers [24,25]. It is important to mention that pure chitosan fibers have rarely been obtained in acetic acid at 90% and 70% [26], but mainly with trifluoroacetic acid (TFA) [27,28] or mixture of TFA and dichloromethane [22,29]. The use of acidic solvent with a low boiling temperature (such as acetic acid or formic acid) allows extraction of excess acid and water, constituting the solvent left after the electrospinning in ambient conditions. Chitosan (CS) fiber production using ionic liquid was also mentioned with 1,1,1,3,3,3-hexafluoro-2-propanol [30].

Generally, it has been shown that fine nanofibers can be produced in chitosan blends with poly (vinyl alcohol) (PVA) [22,25,31,32], gelatin or collagen [2,7,33,34,35,36], silk fibroin [5], polycaprolactone [37,38,39,40] or PET [41], but mainly with poly (ethylene oxide) (PEO) [6,17,18,42,43,44,45,46,47]. Usually, to generate composite fibers, the spinning solutions are obtained by mixing the two polymeric solutions prepared separately in the same solvent. Then, processing of chitosan blends occurs by mono or coextrusion [48]. Core-shell structured PEO-chitosan nanofibers are also prepared, leading to a hollow nanofiber by removing PEO after washing with water [49]. Good spinnability of PEO is recognized, and interaction with chitosan favors the processing [42] in addition to its low toxicity [50].

The chitosan molecular weight, from which nanofibers have been obtained, varies from 85 kg/mol [49] up to 570 kg/mol [29,43,45]; nevertheless, the real molecular weight is often difficult to determine precisely due to the presence of aggregates [19]. Most generally moderate molecular weights in the range of 100 to 200 kg/mol, were used in different solvents. The concentration selected for electrospinning is directly related to the chitosan molecular weight, which imposes the solution viscosity. The adopted polymer concentration is in the range of 20 to 50 g/L in different solvents [18,22,23,26,31]. Due to swelling of the chitosan blend, crosslinking was proposed using glutaraldehyde [25,28] or genipin [51]. Nevertheless, there are no very promising results after those chemical reactions. Therefore, for biomedical application in aqueous mediums, a more direct way to reduce chitosan swelling and prevent chitosan solubilization should be the modification of the intrinsic properties of chitosan, which becomes insoluble in the –NH_2_ form at pH > 6.5. Then, due to the H-bond network established between chitosan chains, no additional chemical reaction is needed [52,53]. The stability is usually tested by the determinations of the degree of solubility and of the degree of swelling in phosphate buffer (PBS) as well as the study of the morphology of fibers by scanning electron microscopy (SEM). The morphological stability is not often discussed in the literature. The use of acidic solvent with a low boiling temperature (such as acetic acid or formic acid) allows extraction of excess acid and water, constituting the solvent left after the electrospinning in ambient conditions. Then, a neutralization step is important to obtain the insoluble chitosan. For this purpose, it was proposed to immerse the nanofiber membrane in 5 M NaOH or 5 M Na_2_CO_3_ aqueous solutions for 3 h at ambient conditions [29], in saturated aqueous solution of Na_2_CO_3_ [23,29], or in 1M NaOH [38], in 1 M K_2_CO_3_ for 3 h at 25 °C [27], or in two solutions of 1M K_2_CO_3_, dissolved in 15 mL of ethanol (70%), and 5M NaOH dissolved in 15 mL of methanol (70%) [54]. Recently, the following method was used to neutralize the chitosan—alkaline ethanol/water (70/30) mixture dissolving K_2_CO_3_ at pH = 12 [52,53].

Besides, it has been described that the CS/hyaluronan (HA) hybrid support serves as an ideal biomaterial to create a three-dimensional (3D) scaffold with adequate strength, high cellular adhesivity, and excellent support for chondrogenesis, preserving the phenotype and enhancing production of type II collagen (with increase of type II/type I collagen ratio) [55]. Data obtained on CS/HA hybrid fibers indicate that materials including HA provide excellent adhesivity for seeded chondrocytes and enhance their biological behavior on the 3D scaffolds with different pore sizes [56].

Then, hyaluronan (HA), also called hyaluronic acid, a natural polysaccharide and a critical component of natural ECM has been widely used in tissue engineering and regenerative medicine [57,58,59,60,61]. These authors produced HA fibers using dimethylformamide (DMF)/water mixed solvents in different volume ratios (0:1, 0.25:1, 0.5:1, and 1:1, respectively) [57]. Nanofibers have been also produced by the electrospinning of HA using a mixture of 25% aqueous ammonium solution and N-methylpyrrolidone in a ratio of 2:1 [58]. Fibers were also obtained using NH_4_OH (25% in water) and DMF in a ratio of 2:1 [59]. Due to difficulties in producing HA fibers, it was proposed to electrospin the blend HA/PEO, giving a core–shell structure [60]. In these conditions, HA fibers are soluble in aqueous medium. The HA nanofibrous membranes electrospun from HA solution in the 1:1 ratio of DMF/water mixed solvent were stabilized by immersion in concentrated hydrochloric acid solution for 10 min [58]. The membrane was stable in the deionized water in a gel state for a short time after being stabilized in HCl solution for ten minutes, but these nanofibers were quite stable in the acid solution for several weeks. This behavior is related to the formation of a physical gel at pH~2.5 in HA solution [62]. These results concerning electrospinning of pure HA were obtained in many solvent conditions but the stability of the fibers in aqueous medium remains low due to the high solubility of HA.

To prevent solubilization of HA, polyelectrolyte complex with chitosan was investigated. Previously, interesting nanofibers were prepared from chitosan/alginate polyelectrolyte complex [63]. Chitosan/PEO and alginate/PEO solutions have been mixed for electrospinning with different volume ratios (20/80, 30/70, 40/60, 50/50, and 80/20). Those nanofibrous scaffolds were able to promote the adhesion and proliferation of cells. This result indicated that nanofibers made of a polyelectrolyte complex may be stable in aqueous medium due to strong electrostatic interactions. Fibers have also been produced by coaxial electrospinning—the alginate solution blended with PEO was used to form the inside (core) of the nanofibers and the chitosan solution mixed with PEO composed the outside [64]. Due to difficulties in electrospinning pure HA, Ma and co-workers proposed blended HA-based systems and successfully electrospun HA solubilized in water (W)/formic acid (FA) (25/75 *w*/*w*) with positively charged chitosan in W/FA (20/80 *w*/*w*) [65] or by mixing HA solution in W/FA/DMF (5.0/2.5/2.5, *w*/*w*) and chitosan solution in W/FA (1/9, *w*/*w*) at the different blending weight ratios from 9/1 to 5/5 [65]. It was shown that the mechanical properties (strength and stiffness) increase for the complex. In addition, it demonstrated a significantly better biocompatibility and higher number of living cells on the surface of the CS–HA compared with CS. Chitosan/hyaluronan hybrid biomaterials proposed in cartilage tissue engineering were formed by wet-spinning of chitosan solubilized in 2% acetic acid immersed in water/methanol (1:1) followed by a calcium solution and coated with hyaluronan [55,56,66,67]. The same technique was proposed to establish medical use for ligament and tendon tissue engineering [68].

In our work, the electrospinning of chitosan-based nanofibers is developed in order to obtain a high yield of chitosan up to pure chitosan nanofibers, which is usually not the case in published papers. Especially, rare methods propose the neutralization of electrospun chitosan nanofibers to get insoluble materials for further application. To produce pure chitosan fibers, two different techniques are used: (1) PEO and CS solutions are mixed to produce nanofibers; (2) powdered PEO is added into chitosan solutions. Then, neutralization and extraction of additives is introduced using alkaline non-solvent conditions. Due to toxicity of the solvents able to be used, the acetic acid in absence of any additive is chosen at the concentration of 0.5 M, corresponding to maximum of chitosan solubility and still having good volatility [69]. The change of the molecular weight and weight ratio of PEO in the chitosan blend are investigated and related with the electrospun nanofiber thickness and physical properties. The mechanical properties of pure chitosan fibers are determined in the dried and wet states [53]. These fiber mats are applied to test chondrocyte adhesion and proliferation, with the results being compared to chitosan film.

After production of chitosan, polyelectrolyte complex (PEC) systems (also referred as hybrid) made of chitosan and hyaluronan are prepared from chitosan and hyaluronan solutions in the same solvent (water/formic acid 50/50 *v*/*v*) at controlled –NH2/–COONa ratios, taking benefit of both polymer relevant properties. The stability of the new materials is controlled by a thermal treatment. In this regard, complex stability is characterized by measuring the solubility, the swelling degrees, and mechanical properties.

## 2. Materials and Methods

### 2.1. Materials

Chitosan (CS) samples from northern cold-water shrimp, *Pandalus borealis,* were a gift from Primex Cy (Batch TM4778, code 42010). Three different molar masses (MW) were used: 102 kg/mol and degree of acetylation determined using ^1^H NMR is DA = 0.12, 500 kg/mol, DA = 0.19 [52], and MW = 160 kg/mol, DA = 0.05 [53]. The hyaluronan sample was purchased from Soliance (Pomacle, France) with an average molecular weight, MW = 540 kg/mol. Poly (ethylene oxide) (PEO) with a different molecular weight MW (300, 1 × 10^3^, and 5 × 10^3^ kg/mol), acetic acid (≥99.7%), formic acid (FA) (ACS reagent > 98%), ethanol, and K_2_CO_3_ were purchased from Sigma-Aldrich (Germany). Deionized water (from EAU, France) was used as solvent to prepare the solutions. Dulbecco’s phosphate buffered saline (DPBS), pH = 7.4, (ref. 14190-094, Lot 2118924) is from Gibco (Paisley, UK). All reagents and polymers were used as received without further purification.

### 2.2. Solution Preparation

Chitosan (CS) solutions were prepared at 5% (*w*/*v*) in 0.5 M acetic acid. These solutions were obtained at room temperature with slow stirring for 4 days to get homogeneous solutions. In the same manner, poly (ethylene oxide) with different MW were solubilized at 5% in 0.5 M acetic acid on a rotating stirrer. Chitosan (CS) solutions were mixed with the solutions of PEO at chitosan/PEO weight ratio (in %) of 95/5, 90/10, 80/20, 70/30, and 60/40. Similarly, the same concentration of CS was blended with powdered PEO. The weight ratios were expressed as weight of chitosan or PEO divided by the total polymer weight for each system tested.

Chitosan and hyaluronan homogeneous solutions were prepared separately at 4% (*w*/*w*) in water/formic acid mixtures (W/FA) at ratios 75/25, 50/50, and 25/75 (*v*/*v*) to obtain stable solutions. In these conditions, the total functional group contents were 0.233 [–NH_2_]/L in chitosan and 0.1 [–COOH]/L in hyaluronan, respectively. Polyelectrolyte complex systems (PEC) are usually very difficult to process due to phase separation related to strong electrostatic interactions. The convenient solvent is essential; hence, W/AF mixture 50/50 *v*/*v* was selected [70]. Subsequently, HA and CS solutions were mixed at different volume ratios corresponding to charge ratios R_C_ = 0.5, 1, 1.8, 2.35, and 3.0 under stirring, getting a homogeneous blend. In order to favor PEC spinnability, the addition of a 4% PEO *w*/*w* solution was needed. Using the same solvent as for the corresponding biopolymer mixture, final contents in PEC/PEO equaling 80/20 and 70/30 (*w*/*w*) were selected such as to preserve a high yield in polysaccharides in the fibers.

### 2.3. Chitosan and PEC Nanofiber Stabilization

Weighted initial chitosan nanofiber mats and films cut in pieces were immersed in an alkaline ethanol/water (70/30) mixture dissolving K_2_CO_3_ at pH = 12 to neutralize the chitosan and get its –NH_2_ form. Further, nanofiber membranes and films were washed with deionized water four times in a day during 3 days until neutral pH was achieved in order to obtain removal of the salts formed from chitosan solutions (potassium acetate), K_2_CO_3_ excess, and PEO. Lastly, the membranes were dried at room temperature for further determination of the swelling capacity or rehydration after a first drying of the materials.

Due to the solubility of HA, the stabilization of the polyelectrolyte complex was performed by thermal treatment. As proposed in the literature, amide linkage is formed between –NH_2_ and –COOH under controlled thermal treatment at a temperature between 80 and 120 °C up to 4 h [71,72,73,74,75]. After testing the kinetic of the reaction at 120 °C, CS and CS/HA complexes under film and fiber mat morphologies were treated at 120 °C for 4 h in air [70].

### 2.4. Casting and Stabilization of Chitosan and PEC Films

Around 1 g of each of the CS and PEC were placed in a Teflon mold of known volume to obtain a uniform polymer film. The probes were stored at room temperature for 3 days until complete evaporation of the solvent. CS films were neutralized, and PEC films were submitted to the thermal treatment as proposed for fibers (Section 2.3). Different samples of a regular shape were taken from the films obtained for future measurements of their mechanical properties and degree of swelling.

### 2.5. Electrospinning

The prepared solutions were placed in a 5 mL plastic syringe fitted with a 21-gauge stainless steel needle. The syringe pump delivers solutions at specified flow rate (model: KDS Legato 200, KD Scientific, Holliston, MA, USA), and vertical electrospinning is performed with an applied voltage around 20 kV between the electrodes using a homemade dual high voltage power supplier (±30 kV, iseq GMBH Germany). Then, the nanofibers were recovered on an aluminum film as a collector and kept at 10 to 17 cm from the tip of the needle. The flow rates vary from 0.02 to 1.5 mL/h. The experiments were carried out at room temperature in a closed Plexiglas^®^ box with relative humidity ranging between 40 to 60%. The produced nanofiber matrices were left in ambient conditions to evaporate excess acid and water prior to further analyses.

### 2.6. Characterization of Nanofibers

#### 2.6.1. Morphology of the Nanofiber Membranes

The SEM analyses of the sample morphology of electrospun nanofiber membranes were observed with a scanning electron microscope (Zeiss ultra 55 SEM FEG, Germany) operating at 3 kV. The nanofibers samples were coated with a 10 nm carbon layer prior to SEM imaging.

#### 2.6.2. Determination of Swelling Capacity

The swelling of the nanofibrous membranes and films was examined in terms of water loss between swollen state in water at neutral pH (or phosphate buffer) and final dried weight at room temperature. The wet swollen samples were weighed after blotting with tissue paper to remove excess surface water ($W_{w}$). Accordingly, the dried samples were also weighed repeatedly until the mass became constant ($W_{d}$). The measurements were carried out three times each. These values correspond to the first swelling after extraction of PEO and neutralization of chitosan or thermal treatment of PEC fibers. The average data were taken for the determination of swelling ratio S, expressed as water content (g) per gram of dried material, using the following equation:

(1) $$S\;=\frac{\left(W_{w}-W_{d}\right)}{W_{d}}$$

where $W_{w}$ (g) is the weight of the swollen nanofibrous mat or film and $W_{d}$ (g) is the weight of the samples after drying at room temperature.

Considering *W_i_* as the weight of the as-spun sample, the difference (*W_i_* − *W_d_*) allows the determination of the eventual degree of the solubility.

#### 2.6.3. Mechanicalharacterization

The measurements were carried out using an ARES-G2 rheometer (TA Instruments, New Castle, DE, USA) equipped with a rectangular geometry, used for axial tension, consisting of two axial clamps that hold the material when the force is applied. Samples were taken from the nanofibrous electrospun matrices and films maintaining a free length/width ratio around 2.69 (suggested value in the rheometer procedure). The dimensions of the rectangular probes used were ~0.7 × 2.5 cm. The results are expressed as the stress (σ) = applied force/section area. In addition, to compare the samples which do not have the same morphology, results of tensile tests are expressed by the reduced force in N/(kg/m^2^), including the mass of the sample divided by its area.

Break tests were performed using the same geometry, starting from a zero-applied force until the material presents a breaking point, with a deformation rate of 0.01 mm/s. The experiments were carried out at constant temperature around 20 °C and a special device was adopted to maintain the relative humidity in the sample environment.

The rheometer also allowed the obtaining of the thickness of the samples by measuring the gap between the two plates when they approach the film or fiber mat as close as possible until the detector perceives zero axial force during compression. This measurement was repeated with a micrometer (Mitutoyo Digimatic micrometer—0 to 25 mm with precision of 0.001 mm), giving very close values. Both techniques used to determine the thickness are in good agreement with a precision of 1 μm.

The dynamo-mechanical analysis (DMA) tests were performed using an initial force applied of 0.02N with deformation between 0.01 and 0.1% strain imposed by the length/width ratio of the sample. For analysis, the storage moduli E observed were normalized taking into consideration the sample weight per unit of surface with the expression:

(2) $$E_{s}\;\left(Specific\;modulus\right)=\;\frac{\;Storage\;Modulus\left(Pa\right)}{\left(\frac{Sample\;\;weight\left(Kg\right)}{Sample\;surface\;\left(m^{2}\right)}\right)}$$

### 2.7. Cell Culture and Cell Development

#### 2.7.1. Cell Culture

For cell culture, the C-20/A4 human chondrocyte cell line [76] was selected and seeded in Dulbecco’s modified Eagle medium (DMEM) supplemented with 10% *v*/*v* of fetal bovine serum (FBS) and a penicillin/streptomycin/glutamine solution at 1% *v*/*v*. Phosphate buffered saline (PBS) solution with a pH = 7.4, measured in the laboratory, DMEM serum-free, and 0.05% Trypsin-EDTA solution were also utilized in cell experiments. All biological reagents were purchased from Gibco Life Technologies (Paisley, UK).

Samples of fiber mats prepared with the blend CS/PEO at a ratio of 80/20 were selected because of their optimal behavior in the electrospinning process, and were cut and weighted for cell culture. Without any other treatment, the nanofiber mats having a regular shape with a surface of ~1 cm^2^, were placed in a Petri dish and washed 2 times with the PBS solution to be subsequently hydrated in the DMEM culture solution for 2 days. After the DMEM solution was extracted from the culture dish, 10 µL of a cell suspension at a concentration of 1 × 10^6^ cell/mL, measured by fluorescence (see Section 2.7.2), were disposed on the fiber mat followed by the addition of 2 mL of complete DMEM. The samples were maintained into a cell incubator (inCu safe, Panasonic) at 37 °C and 5% CO_2_ constant inlet flow during the few days before cell growth quantification, and the culture solution was renewed every 3 days.

The described procedure was followed as well for cell culture on neutralized CS films. Data were compared with the development obtained on chitosan films having the same surface as the fiber mat.

#### 2.7.2. Cell Quantification

Once the incubation time was reached (up to 24 h for cell adhesion and up to 3 weeks for cell proliferation measurements), chondrocytes were detached and suspended in DMEM in order to quantify the number of existing cells on the substrates as a function of time (t). Two protocols were followed considering the final objective, i.e., counting of cell proliferation or cell adhesion.

Cell counting was performed at several times of adhesion and proliferation after cell seeding. The chitosan supports were disposed in a 15 mL tube and carefully washed twice with 1 mL of PBS solution in order to remove remaining DMEM solution and unattached cells. Washing was followed by a detachment step consisting of the addition of 0.5 mL of Trypsin-EDTA 0.05% and vortex agitation at 1000 rpm for 60 s. Further addition of DMEM and PBS washings helped to resuspend the extracted cells.

The final cell suspension was stained with Acridine Orange/Propidium Iodine fluorescent marker (F230001, Logos biosystems, Villeneuve d’Ascq, France) and cell quantification, in cell/mL, was performed on a dual brightfield and fluorescence cell counter (LUNA-FL, Logos biosystems, Villeneuve d’Ascq, France). This technique allows the identification and quantification of the amount of total and living cells, such as to calculate cell viability and also to get information about average cell size.

### 2.8. Atomic Force Microscopy (AFM) Characterization

#### 2.8.1. Substrate Fixing

Substrate samples, covering the majority of the circular surface (9.2 cm^2^) of the culture Petri dish (Techno-Plastic product AG, Switzerland), were selected. UV curing NOA68 (Norland Optical Adhesive 68, Lot 319, Norland Products, INC, Cranbury, NJ, USA), was used to stick the solid substrates to the bottom part of the culture dish. Different adhesion points were created by putting a small amount of the product between the substrate and the dish; NOA68 was left acting for 15 min under UV radiation before AFM tests.

In order to have a reference surface for the adhesion response, a culture dish was treated with a 5 mg/mL Bovine serum albumin (BSA) solution in PBS buffer for 60 min. In such a case, the surface was negatively charged in the presence of the PBS buffer (pH = 7.4).

Before AFM tests, trypsinization was used for cell detachment from a culture flask; a final cell suspension was prepared in complete DMEM.

#### 2.8.2. AFM Measurements

The experiments were performed on a Nanowizard II AFM from JPK Instruments (Berlin, Germany). Soft tipless V-shaped commercial cantilevers MLCT-O (Bruker, France) with a spring constant (k) around 0.01 N/m were used to measure force strength. The spring constant was calibrated following a classical method; first, the sensitivity (~50 nm/V) was found by contact on a rigid surface, then the method of thermal fluctuations [77] was used to find k ~0.01 N/m.

AFM measurements were effectuated applying the single-cell force spectroscopy (SCFS) method. The global strategy consisted of the attachment of an individual chondrocyte cell, which was extracted from its original culture medium. The cantilever was pre-treated with different proteins, allowing the binding of the cell to the cantilever tip. The cantilever functionalization consisted of the use of biotin-BSA; an overnight treatment by incubation at 37 °C, followed by streptavidin for 10 min under the same conditions, and the final step of the treatment involved the immersion of the tips into a biotin-conA solution for 10 min [78,79]. Intermediate cantilever rinsing with PBS between each step was carried out.

The chondrocyte was first captured with the cantilever in 2 mL of serum-free culture medium. Complete culture medium was added, and the cell then approached the chitosan support, which was fixed at the bottom part of the Petri dish. The force set point (F_c_) was selected as 0.5 nN (applied force limit in the normal direction during the contact time) and the cantilever speed was 1 µm/s.

Tests were carried out at two different contact times (60 and 120 s) and two chitosan substrates (a casted film as model and an electrospun nanofiber mat with an average fiber diameter around 100–250 nm) [53]. A reference surface was prepared by coating the plastic of the Petri dish with BSA. It was determined that, under the same buffer conditions, zeta-potential indicates that cells were also negatively charged.

### 2.9. Statistical Analysis

For characterization of films and fibers, the average of experimental results was given with the precision between extreme values. For morphology of nanofibers, the average fiber diameter (AFD) was calculated by a randomly selected diameter of 500 nanofibers from each sample.

Data for adhesion assays in AFM and cell culture were generated at three independent experiments at least. For AFM measurements, each sample was tested at around 15 contact points. All results are reported as mean with standard error of the mean (mean ± SD) as the error bar. The value *p* < 0.01 was considered statistically significant and it was obtained by one-way analysis of variance (ANOVA) using Excel.

## 3. Results and Discussion

During electrospinning, a jet of polymer solution was emitted from the syringe under the action of a strong electric field (involving voltages of up to several tens of kV). This surface-charged jet was accelerated and stretched. The solvent evaporated in the first centimeters of propagation of the jet in the air, leaving space for a nanometric fiber polymer, which was collected on the metallic support. In this process, the selection of the solvent was essential and played an important role in dried fiber morphology.

### 3.1. Pure Chitosan Nanofiber Electrospinning

As chitosan is not able to be processed directly by electrospinning, a water-soluble polymer PEO was added at different CS/PEO ratios. The solutions were prepared in two ways: (1) the CS chitosan solution was added to PEO under powder form allowing the preservation of the initial concentration of chitosan in the mixtures, (2) CS solution was mixed with a PEO solution at a given polymer concentration and molar mass [52].

#### 3.1.1. Experimental Conditions

The optimum conditions to process nanofibers were determined using 0.5 M acetic acid as solvent for the two polymers of given molar masses. To get different CS/PEO ratios for electrospinning, the two mentioned strategies were adopted. Some results are given in Table 1 and Table 2, allowing the proposal of better experimental conditions, i.e., the conditions to obtain continuous bead free fibers. Presence of beads and fibers was attested by microscopy (magnification 50×).

From these results it is concluded that the molar mass (MW) of added PEO (at 12 mg/mL) have to be at least 1 × 10^6^ g/mol when added into 5% CS solution to avoid bead formation and to get continuous fibers (Table 1). When PEO concentration increases in the blend, fibers are obtained even with lower PEO MW (MW = 300 kg/mol). This indicates that MW of both polymers, as well as their respective concentrations, need to be optimized to produce smooth fibers.

To provide evidence of the role of MW chitosan (MW = 102 and 500 kg/mol), several CS/PEO ratios at total polymer concentrations of 4 and 2%, respectively, were prepared. Results are given in Table 2 showing that for MW = 102 kg/mol chitosan, at least 30% PEO is needed to get beadless fibers. In the case of MW = 500 kg/mol, chitosan concentration has to be decreased to 1.4% as it gives a too high viscosity solution. Then, no more fibers were obtained [52].

After complementary experiments, it was concluded that moderate chitosan MW (100 to 200 kg/mol) is required at 4–5% (*w*/*v*) concentration mixed with PEO to increase spinnability in the range of 1000 kg/mol at the same polymer concentration. Then, the best conditions were (1) to mix the two solutions prepared in 0.5M acetic acid at 5% (*w*/*v*) to get better homogeneity of the blend and (2) to adopt polymer weight ratio CS/PEO in the range 80/20–70/30 [53].

#### 3.1.2. Characterization of Chitosan Nanofibers

**Figure 1:**
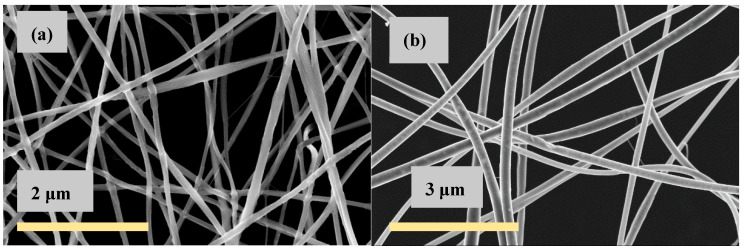
As-spun porous fiber mats produced from (**a**) 80/20 5% CS/PEO solutions, PEO MW = 1000 kg/mol and (**b**) 95/5 5% CS/PEO solutions. CS MW = 160 kg/mol and PEO MW = 5000 kg/mol [53].

The nanofiber mat is used as spun or after neutralization as described in Section 2.3. In this case, it is shown that the fiber morphology (fiber diameters around 150 nm) is perfectly preserved after PEO and salts extraction. It consists of pure chitosan, as demonstrated by nuclear magnetic resonance (NMR) [52,53]. An example of fiber morphology obtained by SEM is shown in Figure 1.

The fiber diameters increase when using a PEO with MW = 5000 kg/mol in relation with the larger viscosity of the blend to be processed [53]. In addition, it was shown that the degree of swelling of chitosan fibers after neutralization, drying, and rehydration depends on the composition of the blend used (around 4 g water/g dried material), increasing when PEO yield in the blend increases (Table 3). Those pure chitosan fibers are perfectly stable in a neutral condition and PBS buffer.

In addition, the density of the fiber mat is determined as well as its thickness. The density observed is nearly constant (0.180 ± 0.02 g/cm^3^).

**Figure 2:**
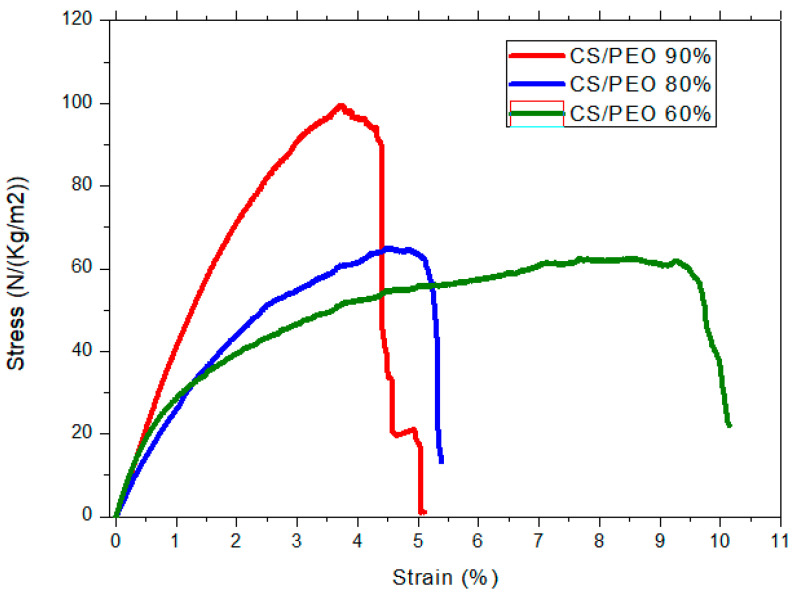
Uniaxial extension for as-spun CS/PEO nanofibers obtained from different composition blends. CS MW = 160 kg/mol and PEO MW = 5000 kg/mol.

The mechanical behavior of as-spun nanofiber mats measured by axial extension in a dried state is shown in Figure 2 for different CS/PEO ratios from blends made of 5% polymer solutions in 0.5 M acetic acid [53].

From these data, it is clear that the increasing PEO content in the nanofiber mat increases fiber elasticity and reduces mat stiffness. In the same manner, higher PEO content in the nanofiber mat implies less CS present in the blend, evidencing the PEO plasticizing effect related to the good compatibility of both polymers. Few values are also mentioned in Table 4 where the stress at break and the strain in % are given as a function of the blend composition on neutralized fiber mats (pure chitosan) in a rehydrated state. In the wet state, it is found that strain at break largely increases compared to the dried state, while the stress at break reduces (compared to Figure 2). The experimental values have the same order of magnitude as soft human tissues [80].

In the same table, the elastic modulus E_S_ of the samples in the initial state and in the wet state after rehydration obtained by DMA shows that the performances decrease by a factor of 2.25 ± 0.03. These results indicate that the material could have a good mechanical behavior as a porous biomaterial stable in aqueous medium and is able to be used in many biological applications, such as wound dressing or cartilage repair.

### 3.2. Complex Chitosan/Hyaluronan Nanofibers

A mixture of oppositely charged polyelectrolytes form a complex stabilized by ionic linkage. This mixture usually forms a coacervate difficult to process with a stability depending on the pH (for weak electrolytes) and external salt concentration. It was important to find conditions to get a homogeneous solution able to be processed by electrospinning.

In our work, after production of chitosan and hyaluronan (not described) fibers by electrospinning, PEC made of chitosan and hyaluronan were prepared from chitosan and hyaluronan solutions in the same solvent at controlled –NH_2_/–COONa ratios, which impose the electrostatic interactions. The stability of the materials obtained was studied on films casted (as a model) from the PEC prepared in the same conditions as for the nanofibers prepared by electrospinning. The use of a film as a model permits easier characterization and development of the methods adopted to optimize the experimental conditions for PEC stabilization. This step is necessary due to the large solubility of HA at neutral pH. The role of thermal treatment on the complex stability was finally characterized by measuring the solubility and swelling degrees and mechanical properties.

#### 3.2.1. Experimental Conditions

After preliminary experiences, CS and CS/HA complexes under film and fiber mat morphologies were treated at 120 °C for 4 h in air conditions for structural stabilization. As proposed in the literature, amide linkages are formed between –NH_2_ and –COOH under controlled thermal treatments at temperatures between 80 and 120 °C, up to 4 h [71,72,73,74,75].

From the weight loss of PEC films during heating, it was observed that the behavior of complexes is similar for the different ratios R_C_ = NH_2_/COONa increasing progressively with the time and slightly with chitosan content. This indicates a larger cross-linkage degree due to H-bonds and probably amide bond formation involving free –NH_2_.

Comparison with chitosan alone shows that the weight loss is higher for free chitosan (18%) than for complexes (~14%) due to lower interaction between chains (thermal treatment of chitosan induces an increase of crystallinity) and a lower degree of reaction with residual formic acid used as solvent [70].

#### 3.2.2. Characterization

In order to optimize the production of nanofibers at high yield in PEC, different experimental conditions were explored. The fibers were obtained from chitosan and chitosan/hyaluronic complex solutions in formic acid/water 50/50 *v*/*v*.

For the electrospinning process, the collector was designed to permit the recovery of the fiber matrix from a metal plate to avoid sticking on the support. For that purpose, aluminum foils cut in cross-section have been chosen as support to remove the mat after processing [70]. In these conditions, the probes for mechanical tests are easy to take out. Good fibers were obtained as soon as the charge ratio reached R_C_ = 1, as shown in Table 5.

Examples of results under the best experimental conditions are given in Table 6 for as-spun PEC/PEO 70/30 nanofiber samples with R_C_ = 2.35 and 3.0. For thermal treatment at 120 °C for 4 h, the weight loss is around 10%. Then, after several PEO and salt extractions with ethanol/water 80/20 *v*/*v*, the samples were dried at room temperature and immerged in PBS at pH = 7.4 to determine solubility and swelling degrees (Table 6).

These data confirm that, for the first time to our knowledge, new biomaterials based on PEC involving HA and CS are obtained which have low degrees of solubility (~10%) and swelling (~3.5%) in PBS at pH = 7.4. Then, it will be convenient for biological applications. The polymer yield after extraction of the 30% PEO included in the blend corresponds to around 70% remaining as indicated in Table 6.

**Figure 3:**
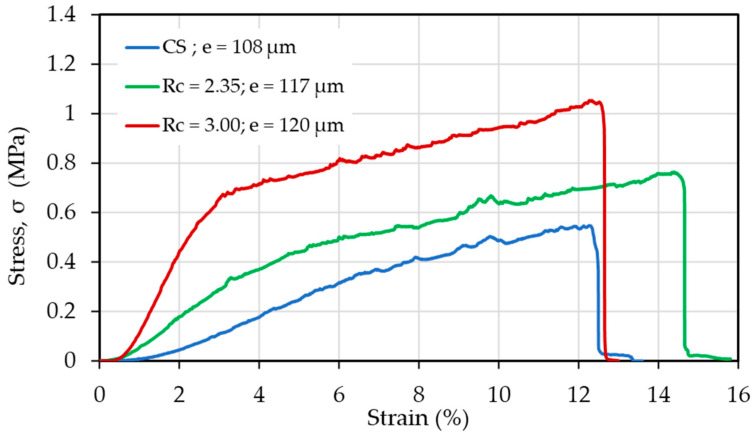
Mechanical behavior of as-spun chitosan and PEC fiber samples in a dried state.

Traction experiments were performed on samples having nearly the same thickness (e in μm) in the absence of thermal treatment (Figure 3). Firstly, it is shown that the PEC fibers are stronger than CS fibers as soon as R_C_ is higher than 1.8, with a relatively large strain at break probably connected with the presence of PEO. The stress at break is increasing directly as a function of the chitosan content.

The application of the thermal treatment, at 120 °C for 4 h, confirms that it stabilizes the material by decreasing the aqueous medium solubility and swelling degree and increasing the mechanical performances. This effect is based on the hypothesis of amide and H-bond formation involving –NH_2_ and –COOH functions. Over a charge ratio larger than 1.8, the swelling and solubility are stabilized at pH > 7 after thermal treatment.

From these results, it is confirmed that the insoluble material after extraction and thermal treatment consists of the HA/CS complex, allowing us to take advantage of the specific properties of the two important biological polymers, HA and CS.

### 3.3. Application in Tissue Engineering

In the following, our objective was to prove the validity of chitosan nanofiber mats to improve chondrocyte cell adhesion and proliferation for tissue engineering applications. For that purpose, adhesion force measurements were determined by AFM and cell development was investigated, comparing chitosan homogeneous films and nanofiber mats. Experiments were performed using chitosan MW = 160 kg/mol and DA = 0.05 solubilized at 5% (*w*/*v*) in 0.5 M acetic acid.

#### 3.3.1. Interaction of Cells/Chitosan by AFM

To demonstrate the advantage of electrospun chitosan nanofibers on cell adhesion, force measurements at the nanoscale provided by AFM were performed. The results were compared with the adhesion response on chitosan films, as well as on a BSA-coated Petri dish surface as reference.

**Figure 4:**
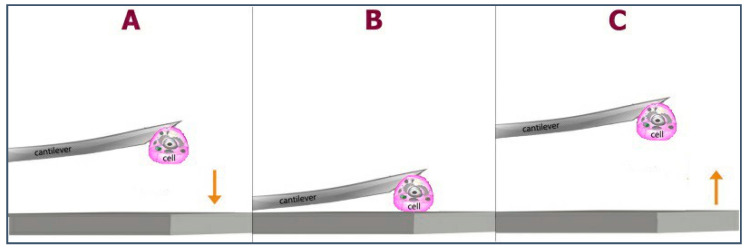
Global strategy for the cell adhesion measurements. (**A**) Approach. Chondrocyte is attached to the cantilever and approaches the chitosan substrate at constant velocity. (**B**) Contact. Chondrocyte is in contact with the substrate during the contact time (t_c_) under an applied contact force (F_c_). (**C**) Retraction. The cantilever is retracted, and the cell interaction response is obtained.

AFM allowed the characterization of normal cell-substrate interaction, such as cell adhesion and detachment, using the single-cell force spectroscopy (SCFS) method to perform adhesion measurements [78,79]. SCFS consists of the immobilization of a single living cell on a cantilever and the measurement of the interaction forces between the cell and a surface, i.e., the chitosan substrates. For this approach, the cell attached to the cantilever is pushed until contact with the substrate, as depicted in Figure 4, allowing for direct measurement of cell-surface adhesion force.

**Figure 5:**
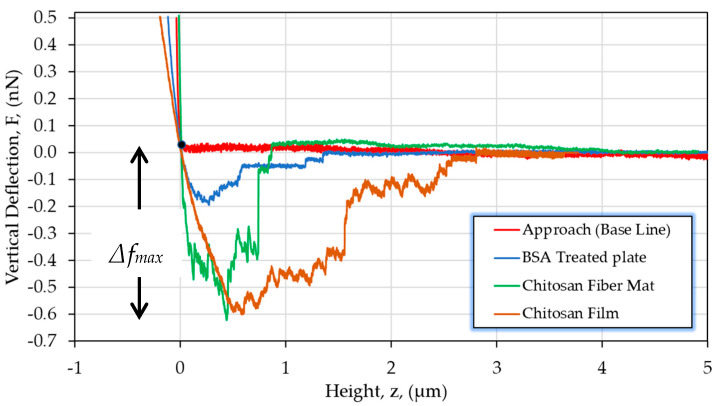
Comparative response for a chondrocyte detachment on chitosan substrates (film and fiber mat) and coated Petri dish. The point (0,0) on the curve F vs. z represents the cell-substrate contact point and *Δf_max_* the maximal normal force observed for the detachment response. Retraction velocity of 1 µm/s and data shown for a contact time (t_c_) = 60 s.

The curves corresponding to the approach and retraction processes are presented in Figure 5. During retraction, complete detachment of the cell occurs and vertical force F(nN) of the cantilever is represented versus piezo height (z). During the contact between the chondrocyte and the surface, both indentation and adhesion interactions are suggested to occur.

When the retraction region of the curves was analyzed, we were able to determine adhesion forces required to break adhesion bonds. This is directly related to the adherent protein distribution among the cellular membrane that, especially in chondrocytes, mediates the capacity of the cell to make specific contact with the ECM [81].

**Figure 6:**
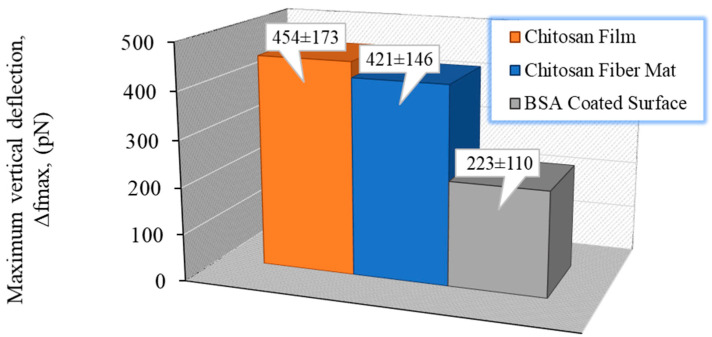
Distribution of maximum vertical force (*Δf_max_*) for the two substrates studied—chitosan film and chitosan nanofibers compared to the reference BSA-coated surface (significant difference found, *p* < 0.01), for a contact time of 60 s. Values presented as mean ± SD.

The beginning of the detachment process is set by the highest vertical deflection value (*Δf_max_*). This peak can be associated with the cell-substrate assembly links being stretched at the same time and the point where they start to break. This stage is considered as the more representative part of the detachment response. The distribution of *Δf_max_*, comparing the chitosan film and the electrospun mat for a given contact time of 60 s, is shown in Figure 6.

From these results, a higher maximal normal deflection is observed when a single chondrocyte interacts with a compact surface (the chitosan film) in contrast with the porous fiber mat for which the *Δf_max_* values are slightly smaller. Even though the molecular basis of chondrocyte adhesion is not fully understood, certain cell-surface receptors are known to be present and mediate interactions between chondrocytes and specific ECM components [82,83]. Once chondrocytes touch the substrate, all cell membrane–substrate interactions (specific or not) are expressed. In this manner, the observed adhesion response can be explained considering the quantity of cell–substrate bonds that could be formed in the larger film surface in contact with the cell membrane (hemispherical shaped) during a given contact time. Our results allow us to conclude that for both chitosan substrates used, the adhesion is favored compared to a negative BSA-coated surface; a difference that involves H-bond and electrostatic loose contributions between chondrocyte and chitosan.

#### 3.3.2. Cell Development

Polymeric nanofibers that mimic the structure and function of the natural ECM have received interest as potential scaffolding materials for regenerative medicine. With this aim, the prepared chitosan nanofibers showing good compatibility with chondrocytes were used as substrate for chondrocyte development.

After 14 days of culture, around 10 times more living cells were found to be attached to the biopolymeric fibrous substrates. Fibers made of pure chitosan (neutralization step followed after electrospinning) favor proliferation of chondrocytes in contrast to the chitosan film. It is also shown that, for the first step of adhesion, the adhered cell density is larger on fibers compared with film in relation with the larger area available for cell adhesion for the same material surface (~1 cm^2^), but with larger porosity and lower density.

**Figure 7:**
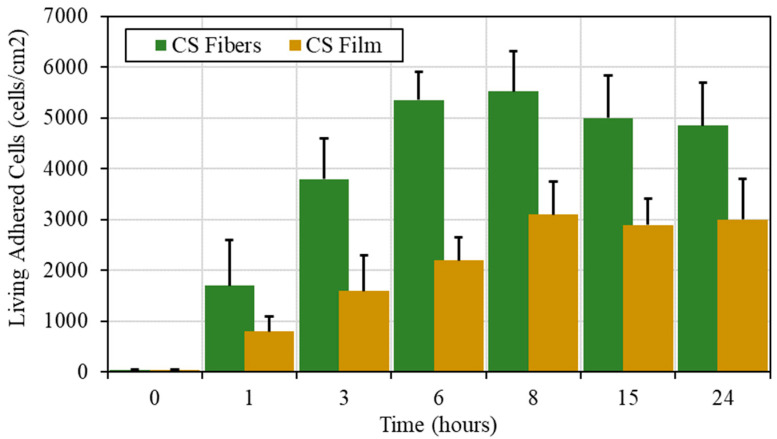
Cell adhesion kinetics of chondrocytes, presented as density of living cells as a function of seeding time, on chitosan fibers produced from CS/PEO blends using PEO MW = 5000 kg/mol compared to the adhesion response on CS film. Error bars represent mean ± SD; n = 3.

For both morphologies, it is shown that the number of living adhered chondrocytes increases as a function of seeding time, as shown in Figure 7.

From one side, cells were observed to develop more efficiently on fibers, probably due to a larger accessible area and high substrate porosity compared to the chitosan films. This result is in agreement with the adhesion force determined previously on single cell interaction with chitosan, where the morphology has a negligible influence in short time observations (60 s). From the other side, a slight decrease is observed at ~8 h after cell seeding on fibers. This behavior could be related to entrapment in the porous fiber mat causing a lower cell detachment yield (i.e., cell quantification).

Cell viability was obtained, indicating a good adaptation of chondrocytes with the chitosan substrate proposed and a large viability when the fraction of living cells is determined at more than 80%, being higher on nanofibers.

**Figure 8:**
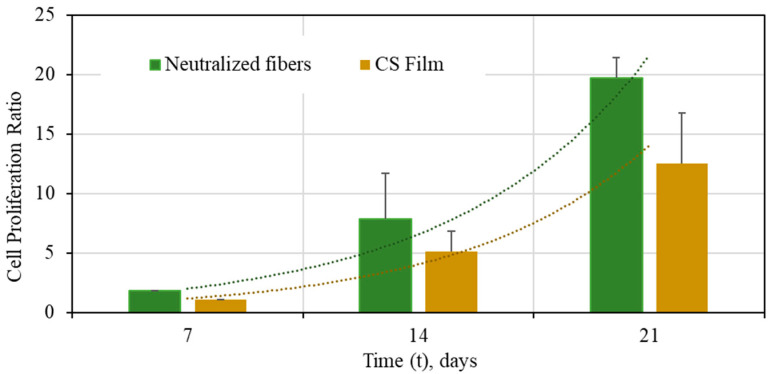
Cell proliferation ratios for chondrocytes on CS/PEO fibers produced from PEO, MW = 5000 kg/mol: pure chitosan fibers (■) and CS films (■). Error bars represent mean ± SD; n = 4 and n = 3 for fiber mats and CS films, respectively.

As presented in Figure 8, chondrocyte population grows as a function of time. A significant difference (*p* < 0.01) for cell proliferation was found comparing CS films and fibers. The reason could be that the cells on the cast films reached confluency before and started detaching, whereas those on the electrospun fibers kept on growing as the available surface was larger [82]. To conclude, application of chitosan nanofibers in tissue engineering allows us to obtain a larger number of adhered cells, a larger fraction of living cells, and a larger degree of proliferation compared with homogeneous films. Additionally, this support is highly porous, with good mechanical properties even in aqueous medium with good biocompatibility.

## 4. Conclusions

This paper describes the main experimental results obtained from electrospinning of chitosan and chitosan/hyaluronan complex to produce nanofiber mats. This process needs the presence of PEO blended with the polysaccharides to allow good spinnability. Polymer concentrations and solvent nature were optimized to get nanofibers free of beads using nontoxic solvent able to be evaporated easily after electrospinning. Acetic acid (0.5 M AC) for pure chitosan and formic acid/water (W/AF 50/50) for chitosan/hyaluronan polyelectrolyte complex were selected. Fibers with diameters in the range 100 to 250 nm free of PEO and solvent traces were stable in biological medium (phosphate buffer pH = 7.4). For the first time, stable pure chitosan and CS/HA complex nanofibers were produced with good stability in PBS buffer. For that purpose, chitosan fibers were neutralized in alkaline non-solvent and CS/HA fibers were thermically treated (120 °C, 4 h) to favor H-bond network formation. Such complex nanofibers were produced for the first time, allowing us to take advantage of the two biologically active polymers for medical applications.

Considering possible applications in cartilage repair, chondrocyte cells were selected to test chitosan/cells interactions and development. With this aim, by the SCFS method, a single chondrocyte was tested in interactions with chitosan. Adhesion force shows the advantage of chitosan compared to a BSA-coated surface, probably related to specific cell/CS interactions. This force is slightly higher for chitosan film compared to fiber mat due to the flat contact with the available surface in a short contact time. The same materials were selected to study the adhesion and proliferation of chondrocytes for 21 days. In relation with the available surface, experiments indicated higher cell viability and proliferation on nanofiber mats. These results on cell development demonstrate that chitosan nanofiber porous mat is a potential material for cartilage repair applications. This work will be further developed to analyze the influence of HA introduced in the complex fibers for chondrocyte development and cell phenotype preservation.

## Figures and Tables

**Table 1:** Composition of electrospun solutions obtained from powdered poly (ethylene oxide) (PEO), with different molecular weights, added into chitosan (CS) (molecular weight (MW) = 102 kg/mol) in 0.5 M acetic acid solution [52].

CS (mg/mL)	PEO (mg/mL)	PEO MW (kg/mol)	CS/PEO (*w*/*w*) %	Electrospun Products
50	12.0	100	80/20	Beads
50	12.0	300	80/20	Beads
50	12.0	1000	80/20	Fibers
50	12.0	5000	80/20	Fibers
50	12.0	8000	80/20	Fibers
50	12.0	300	80/20	Beads
50	20.0	300	70/30	Fibers
50	30.0	300	60/40	Fibers
50	40.0	300	55/45	Fibers

**Table 2:** Electrospun blends of chitosan having different molecular weights with powdered PEO (1000 kg/mol) added [52].

CS (mg/mL)	PEO (mg/mL)	CS-MW (kg/mol)	CS/PEO (*w*/*w*) %	Electrospun Products
35.8	4.2	102	90/10	Beads
32.0	8.0	102	80/20	Fibers + beads
28.6	11.4	102	70/30	Fibers
13.5	1.6	500	90/10	Fibers + beads
12.8	3.4	500	80/20	Fibers + beads
12.3	4.9	500	70/30	Fibers + beads
11.2	7.8	500	60/40	Beads

**Table 3:** Degree of rehydration of nanofibers produced at different CS/PEO ratios after neutralization, extraction of PEO (*M*W = 5 × 10^3^ kg/mol), and drying [53].

System	Fiber Mat Thickness (mm)	Chitosan Weight Concentration Ratio	Density (g/cm^3^)	Rehydration Degree (g/g)
Blend of CS and PEO 5% Solutions	0.128	95	0.173	3.58
	0.122	90	0.163	3.71
	0.102	80	0.200	3.95
	0.115	70	0.164	4.19

**Table 4:** Experimental *E_s_* values obtained by DMA on as spun and wet fiber mats after extraction of PEO (MW = 5 × 10^3^ kg/mol), drying and rehydration. Uniaxial stress/strain properties on wet state are joined [53].

System	[CS] %	$Es\left(\frac{MPa}{kg/m^{2}\;}\right)$ Initial State	$Es\left(\frac{MPa}{kg/m^{2}\;}\right)$ Wet State after Rehydration	$\frac{Einitial}{Eswollen}$	*σ_Break_*		*ε_Break_*
					MPa	$\frac{N}{kg/m^{2}\;}$	%
5% CS/PEO solution	70	939	421	2.23	0.42	18	44.9
	80	1490	652	2.28	1.43	40	46.0
	90	1370	602	2.28	1.19	47	31.9

**Table 5:** Relation between polyelectrolyte complex system (PEC) composition and electrospinning results.

Charge Ratio NH_2_/COOH	Weight Ratio NH_2_/COOH	Electrospun Products
0.5	0.21	Fibers, few beads
1.0	0.42	Fibers
1.8	0.77	Fibers
2.35	1.0	Fibers
3.0	1.26	Fibers

**Table 6:** Weight loss for thermal treatment at 120 °C for 4 h and stability of nanofibers for R_C_ = 2.35 and 3.

R_C_	Weight Loss (%) after TT	Remaining Polymer (%) after EtOH/H_2_O Washing	Swelling Degree (gH_2_O/g) at pH 7.4	Solubility (%) at pH = 7.4
2.35	9.8 ± 2.5	69.8 ± 8.1	3.3 ± 0.3	12.2
3.0	10.9 ± 0.4	73.79 ± 0.18	3.7 ± 0.5	13.9

## Data Availability

Not applicable.
